# 430. Strategies for Prevention of COVID-19 Transmission in Hospitals

**DOI:** 10.1093/ofid/ofab466.630

**Published:** 2021-12-04

**Authors:** Wooyoung Jang, Bongyoung Kim, Eu Suk Kim, Kyoung-Ho Song, Song Mi Moon, Myung Jin Lee, Ji Young Park, Ji-Yeon Kim, Myoung Jin Shin, Kurt Stevenson, Hong Bin Kim

**Affiliations:** 1 Hanyang University College of Medicine, Seongdong-gu, Seoul-t’ukpyolsi, Republic of Korea; 2 Department of Internal Medicine, Seoul National University College of Medicine, Seoul, Korea, Seoul, Seoul-t’ukpyolsi, Republic of Korea; 3 Department of Internal Medicine, Seoul National University College of Medicine, Seoul National University Bundang Hospital, Seoul, Seoul-t’ukpyolsi, Republic of Korea; 4 Division of Infectious Diseases, Department of Internal Medicine, Inje University Sanggye-Paik Hospital, Seoul, Korea, Seoul, Seoul-t’ukpyolsi, Republic of Korea; 5 Seoul Natkional University Bundang Hospital, Seongnam, Kyonggi-do, Republic of Korea; 6 Seongnam Citizens Medical Center, Seongnam-si, Kyonggi-do, Republic of Korea; 7 Seoul National University Bundang Hospital, Sungnam, Kyonggi-do, Republic of Korea; 8 The Ohio State University College of Medicine and College of Public Health, Columbus, Ohio

## Abstract

**Background:**

Infection control measures against the coronavirus disease 2019 (COVID-19) within a hospital often rely on expert experience and intuition due to the lack of clear guidelines. This study surveyed current strategies for the prevention of the spread of COVID-19 in medical institutions.

**Methods:**

Upon systematic review of the guidelines at the national level, 14 key topics were selected. Six hospitals were provided an open survey that assessed their responses to these topics between August 11 and 25, 2020. Using these data, an online questionnaire was developed and sent to the infection control teams of 46 hospitals in South Korea. The survey was conducted between January 31, 2021, and February 20, 2021.

**Results:**

All 46 hospitals responded to the survey, and 24 hospitals (52.2%) had treated 100 or more cases of COVID-19. All hospitals operated screening clinics, and the criteria were respiratory symptoms (100%), fever (97.8%), and epidemiological association (93.5%). It was found that 89.1% (41/46) of hospitals allowed symptomatic patients to visit their general outpatient clinics if fever or respiratory symptoms were not associated with COVID-19. Most hospitals (87.2%; 34/39) conducted polymerase chain reaction (PCR) tests for all hospitalized patients. Moreover, 76.1% (35/46) of hospitals implemented preemptive isolation policies for hospitalized patients, of which 97.1% (34/35) were released from isolation after a single negative PCR test. A little over half of the hospitals (58.7%; 27/46) treated patients that met the national criteria for release from isolation but consistently had positive PCR results. Of these hospitals, 63% (17/27) used N95/KF94 masks, and 40.7% (11/27) used surgical masks without other personal protective equipment for treating them. Most hospitals (76.9%; 20/26) accommodated them in shared rooms when the cycle threshold value of the PCR test was more than a certain value (34.6%; 9/26), or after a certain period that satisfied the national criteria (26.9%; 7/26). Finally, 76.1% (35/46) of hospitals performed emergency procedures or operations on suspected patients.

Table 1. Screening and selective treatment policy to prevent COVID-19 patients from entering the hospital

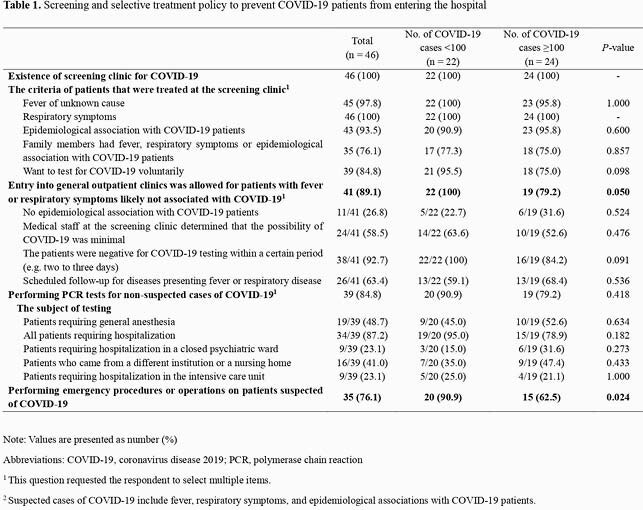

**Note:**

Values are presented as number (%) Abbreviations: COVID-19, coronavirus disease 2019; PCR, polymerase chain reaction 1 This question requested the respondent to select multiple items. 2 Suspected cases of COVID-19 include fever, respiratory symptoms, and epidemiological associations with COVID-19 patients.

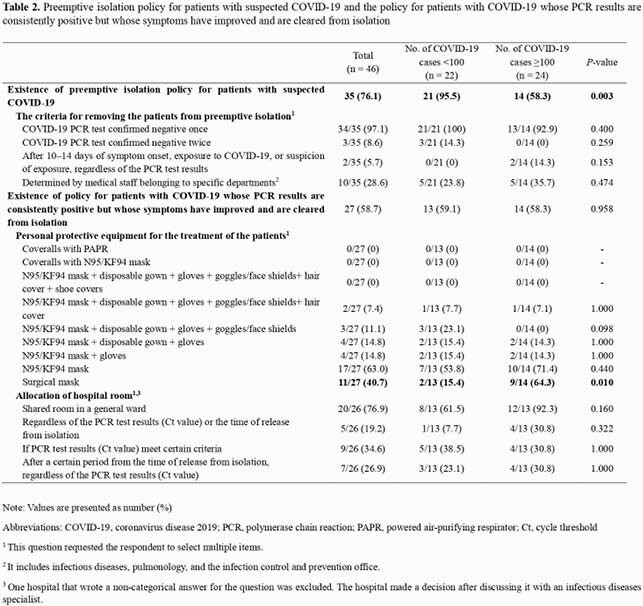

**Note:**

Values are presented as number (%) Abbreviations: COVID-19, coronavirus disease 2019; PCR, polymerase chain reaction; PAPR, powered air-purifying respirator; Ct, cycle threshold 1 This question requested the respondent to select multiple items. 2 It includes infectious diseases, pulmonology, and the infection control and prevention office. 3 One hospital that wrote a non-categorical answer for the question was excluded. The hospital made a decision after discussing it with an infectious diseases specialist.

**Conclusion:**

Various guidelines were being applied by each medical institution, but there was a lack of an explicit set of national guidelines to support them.

**Disclosures:**

**All Authors**: No reported disclosures

